# De Novo Postinfectious Glomerulonephritis Secondary to Nephritogenic Streptococci as the Cause of Transplant Acute Kidney Injury: A Case Report and Review of the Literature

**DOI:** 10.1155/2018/2695178

**Published:** 2018-05-31

**Authors:** Alexander Bullen, Mita M. Shah

**Affiliations:** Division of Nephrology-Hypertension, University of California San Diego, San Diego, CA, USA

## Abstract

Acute kidney injury is common among kidney transplant recipients. Postinfectious glomerulonephritis secondary to nephritogenic streptococci is one of the oldest known etiologies of acute kidney injury in native kidneys but rarely reported among kidney transplant recipients. This report is of a biopsy-proven case of acute kidney injury in a renal allograft recipient caused by de novo poststreptococcal glomerulonephritis.

## 1. Introduction

Acute kidney injury (AKI) is common in kidney transplant recipients (KTR) [1] and it has been identified as a risk factor for graft failure [1, 2] and death with a functioning transplant [2]. Postinfectious glomerulonephritis (PIGN) secondary to nephritogenic streptococci is a well-recognized cause of native kidney AKI. In a seminal paper in 1812, Wells detailed the complications of scarlet fever and described this glomerulonephritis as the “dropsy that follows scarlet fever” [3]. He noted that it mainly occurred in children, approximately three weeks after a mild fever and it consisted of edema starting in the face, decreased urine output, red urine, nausea, and vomiting, which would “recede after no long stay” [3].

PIGN secondary to nephritogenic streptococci has a heterogeneous clinical presentation ranging from asymptomatic to nephrotic syndrome [4]. Diagnosis is based on clinical findings in addition to documentation of a recent GAS infection by positive throat or skin culture or serologic tests, such as anti-streptolysin O (ASO) antibodies [5].

Few reports of PIGN secondary to nephritogenic streptococci as the cause of AKI in KTR are currently available in the literature. We present a biopsy-proven rare cause of AKI in a KTR as PIGN secondary to nephritogenic streptococci.

## 2. Case Presentation

The patient was a 45-year-old Hispanic male who had end-stage renal disease of unknown etiology, hypertension, and hyperlipidemia. His HLA typing was A 2,- B 7, 35, Cw 4, 7, DR 4,- DQ 8,-. His donor was a 46-year-old Hispanic female with history of hyperlipidemia with a measured 24-hour urine creatinine clearance of 151 ml/min. Her HLA typing was A 2,31, B 35,44, Cw 4,5, DR 4,-, DQ 7,8. The patient had been on intermittent hemodialysis for two years prior to undergoing living related kidney transplant. Induction therapy consisted of basiliximab and solumedrol. Maintenance therapy was with tacrolimus, mycophenolate mofetil, and prednisone. His two-year course after transplant had been unremarkable, with a baseline serum creatinine of 1.5–1.7 mg/dL (134–150 *μ*mol/L), without proteinuria or hematuria.

Two years after transplant he presented to the renal transplant clinic with complaints of lower extremity edema that had appeared over the previous three days. He stated he had experienced a flu-like illness a week prior. In addition, he admitted to inadvertently taking tacrolimus 1 mg q12h, rather than his prescribed dose of 3 mg twice a day for almost one month. He had corrected the dose approximately 3 weeks prior to presentation. On examination, he was normotensive and afebrile. Cardiovascular and respiratory examinations were normal. He had periorbital edema and 6 mm pitting edema in lower extremities. He did not have graft tenderness or bruit.

Laboratory data was remarkable for creatinine of 2.2 mg/dL (194 *μ*mol/L). Urinalysis showed moderate blood and 3+ protein (previously no proteinuria), urinary sediment of more than 50 red blood cells (RBCs), 11–20 white blood cells (WBCs) per high power field (HPF), and urine protein/creatinine ratio of 8.2 g (previously 100 mg). Tacrolimus trough was 4.9 ng/mL.

Due to acute kidney injury, proteinuria, and hematuria in the setting of suboptimal immunosuppression, there was a high concern for acute rejection versus rapidly progressive glomerulonephritis perhaps due to recurrence of the unknown primary disease. Renal ultrasound and a renal biopsy were ordered. Given the risk of acute rejection due to inadvertent medication noncompliance, prednisone was increased from 10 mg daily to 50 mg daily, tacrolimus was increased from 3 mg twice a day to 5 mg twice a day, and mycophenolate mofetil was increased to 1500 mg twice a day. Of note, BK virus and donor specific antibodies were negative a month prior.

The renal US was negative for hydronephrosis or calculi. Three days later, a biopsy was performed.

**Figure 1:**
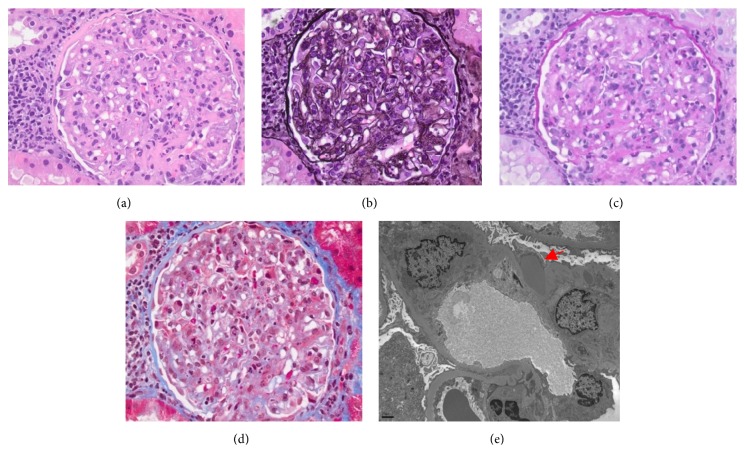
(a) Light microscopy. Hematoxylin and stain. Significant endocapillary proliferation with neutrophils and swollen endothelial cells. (b) Jones stain with occasional double contour. No evidence of significant crescents noted. (c) Periodic acid-Schiff stain revealed mild mixed interstitial inflammation with lymphocytes, plasma cells, and neutrophils. (d) Trichrome stain with focal mild interstitial fibrosis and tubular atrophy. (e) Electron microscopy. Large subepithelial electron dense deposits (arrow) consistent with “humps”.

Preliminary biopsy report was consistent with postinfectious glomerulonephritis (Figure 1). Due to the recent infection, anti-streptolysin O (ASO) antibodies, C3, and C4 were ordered. Since initial biopsy did not have any glomeruli for immunofluorescence (IF), he was scheduled for repeat biopsy.

In the interim, C3 and C4 were reported. C3 was low at 59 mg/dL with a normal C4 at 35 mg/dL (Table 1). Tacrolimus trough was 8.6 ng/mL.

**Figure 2:**
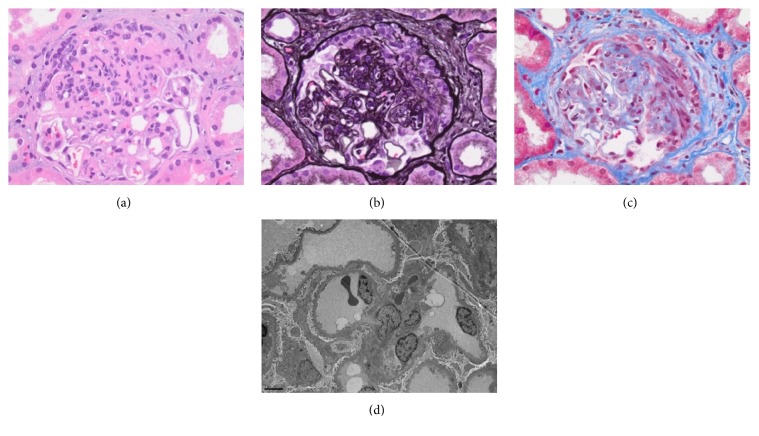
(a) Light microscopy. Hematoxylin and Eosin stain with decreased endocapillary proliferation of neutrophils with some karyorrhectic debris and moderate crescents. (b) Jones stain with an early fibroepithelial crescent. (c) Trichrome stain with crescent and focal mild interstitial fibrosis and tubular atrophy. (d) Electron microscopy. Minimal residual subendothelial electron dense deposits but no evidence of large subepithelial electron dense deposits “humps”.

Second kidney biopsy one week later revealed minimal residual subendothelial electron dense deposits, but no evidence of large subepithelial electron dense deposits (Figure 2). IF showed nonspecific patchy staining with C3 in glomeruli and some tubules. All other reagents were negative, including C4d in peritubular capillaries, BK, and SV40 in tubular cells. There is no evidence of cell-mediated or antibody mediated glomerulonephritis. Overall, biopsy was consistent with resolving postinfectious glomerulonephritis. Anti-streptolysin O (ASO) was elevated at 603 IU/mL (Table 1), highly indicative of* Streptococcus* being the causative agent.

**Figure 3:**
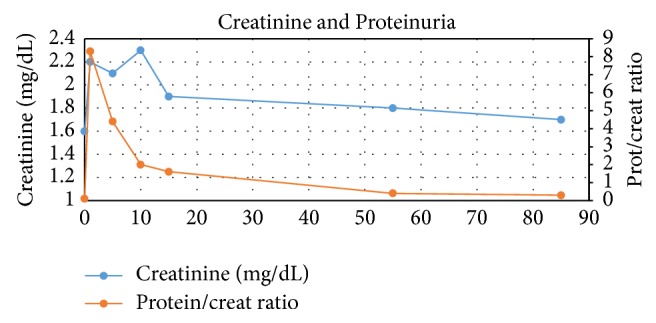
Creatinine and spot urine protein/creatinine ratio during clinical course.

Given that his AKI did not appear to be due to rejection, tacrolimus was decreased back to his basal dose of 3 mg twice a day and prednisone was tapered to 10 mg daily. Fluid management was achieved with furosemide. He was not prescribed any antibiotics. A month later, creatinine had decreased to 1.9 mg/dL (168 *μ*mol/L), and in a 3-month period, it had returned to baseline and proteinuria and hematuria had completely resolved (Figure 3 and Table 1).

## 3. Discussion

AKI is common in renal allograft recipients [1] due to multiple risk factors including single kidney, use of calcineurin inhibitors, and increasing use of marginal donor kidneys, among others. AKI in this population has been identified as a risk factor for graft failure [1, 2] and death with a functioning transplant [2]. Infections are the second most common cause of death in kidney transplants recipients due to multiple factors including immunosuppressed status, donor-derived infection, and underlying comorbidities [6]. CMV disease, Epstein-Barr virus infection, and BK polyomavirus infection are among the most common infections in kidney transplant recipients [7]. Glomerular disease because of infection has been described in KTR in the case of cryoglobulinemic and noncryoglobulinemic membranoproliferative GN secondary to hepatitis C; however, there are only seven reported cases of postinfectious glomerulonephritis [8], with only one reported case to our knowledge of poststreptococcal glomerulonephritis occurring in a pediatric transplant recipient [9]. Three cases were due to* Staphylococcus aureus*, none of which had recovery of their graft function and one died.

PIGN secondary to nephritogenic streptococci is caused by nephritogenic strains of group A beta-hemolytic* Streptococcus*, which cause skin and throat infections. Two of the primary antigens are the nephritis-associated plasmin receptor (NAPIr) [10] and the streptococcal pyrogenic exotoxin B (SPEB) [11]. They both activate the alternative complement pathway [12]. The plasmin-binding capacity of NAPIr plays a key role in the immune complex deposition and local inflammation [13]. SPEB is found in the subepithelial electron dense deposits (humps) which are a characteristic finding of this disease [14]. Over thirty years ago, HLA-DRw4 was found to be associated with increased susceptibility to poststreptococcal glomerulonephritis [15]. Currently, genomics studies with microarray technology and proteomic analysis are being performed to better understand GAS pathogenesis and thus develop targeted therapies [16].

Although poststreptococcal glomerulonephritis is typically thought of as a disease affecting children, it has a bimodal distribution, also affecting patients older than 60 years of age. The clinical spectrum is varied, ranging from asymptomatic disease with microscopic hematuria to acute nephritic syndrome. Patients develop nephrotic range proteinuria, edema, and acute kidney injury. Laboratory studies reveal low C3 and CH50 early in the disease course with typically normal C4 [17, 18]. Nonetheless, there are some cases with low C4, suggestive of activation of classical pathway as well [19].

Some have postulated that PIGN is a self-limiting form of C3 glomerulopathy [20], based on the presence of low levels of C3. However, it is important to denote some key differences between the two diseases. First, in accordance with the hypothesis that C3 glomerulopathy is secondary to an abnormality in the alternative complement pathway, there is bright C3 staining and lack of significant immunoglobulin staining, whereas in PIGN there is bright staining of both C3 and IgG [21]. It is important to note that there are the so called cases of “atypical” PIGN, in which the IgG may not be present in the IF and clinically, there is persistent hematuria and proteinuria and sometimes progression to end-stage renal disease. When Sethi et al. evaluated eleven of such cases; they found an abnormality in the alternative pathway, leading them to speculate that those cases represented undiagnosed C3 glomerulonephritis [22]. Another key difference is found in the electron microscopy where in C3 glomerulopathy, there are mesangial, subendothelial, or sometimes subepithelial or intramembranous deposits which are lobular and amorphous [21]. However, in PIGN, one typically finds clearly demarcated subepithelial humps. Furthermore, C3 glomerulopathy tends to be a progressive disease [23] and it is known to recur in transplant kidneys [24, 25] and in the patient described in this case, the C3 levels returned to normal and he has remained with stable renal function without proteinuria, hematuria, or hypocomplementemia for the past five years. Certainly, as the C3 glomerulopathy consensus report noted in 2013 [26] and the study from Sethi et al. suggests [22], further investigation for C3 glomerulopathy is warranted if any atypical clinical or histological features are present.

De novo acute postinfectious glomerulonephritis is not a commonly reported etiology of AKI in KTRs, but in the few cases that have been reported, some peculiarities have been noted [8]. First, a heterogeneous etiology of bacterial, fungal, viral, and parasitic infections triggering an acute glomerulonephritis is sometimes seen in transplant patients. Second, when the glomerulonephritis was triggered by etiologies other than* Streptococcus*, such as* Staphylococcus* and* E*.* coli*, the most common urinary pathogen in KTRs [9], patients required renal replacement therapy. Interestingly, of the three cases described by Moroni et al., none were caused by* Streptococcus*. Plumb et al. also reported three cases of PIGN caused by* S*.* aureus*, CMV infection, and a presumed urinary tract infection [27]. It is important to note that all the cases they reported of PIGN were in patients with type 1 DM, leading to the speculation that these patients may be at higher risk of developing this glomerular disease as others have suggested [28]. None of the cases that Plumb et al. reported were caused by nephritogenic streptococci. To our knowledge there is only one other case of posttransplant acute PIGN secondary to nephritogenic streptococci in the literature and it was in a 12-year-old male, 1 year after receiving a transplant [9].

Treatment for this disease is mainly supportive, although methylprednisolone and even plasmapheresis have been used, especially when caused by* S. aureus* and* E. coli* [19, 29]. Unfortunately, of the seven cumulative cases reviewed by Moroni et al., four ultimately were restarted on dialysis and one died [8], illustrating the severity of this disease.

## 4. Conclusions

This case reveals the importance of having a prompt and thorough evaluation of acute kidney injury. Although the most common etiologies of AKI in KTRs are calcineurin inhibitor toxicity, recurrence of the primary disease, and acute rejection, relatively uncommon entities such as PIGN secondary to nephritogenic streptococci may cause acute kidney injury.

## Figures and Tables

**Table 1:** Laboratory parameters during clinical course.

Day	0	1	5	10	15	55	85
Creatinine (mg/dL)	1.6	2.2	2.1	2.3	1.9	1.8	1.7
Protein/creat ratio	0.1	8.3	4.4	2	1.6	0.4	0.2
Urine RBCs	0–2	0–2	>50	21–50	11–20	11–20	0–2
Urine WBCs	0–2	0–2	11–20	11–20	11–20	3–5	0–2
C3 (mg/dL)^*∗*^			59		93		146
C4 (mg/dL)^*∗∗*^			34		36		42
ASO Ab^*∗∗∗*^			603				359

^*∗*^C3 normal range: 90–180; ^*∗∗*^C4 normal range: 10–40 mg/dL; ^*∗∗∗*^ASO Ab = anti-streptolysin O antibodies.
